# Perceptions about the accessibility of healthcare services among ethnic minority women: a qualitative study among Arab Bedouins in Israel

**DOI:** 10.1186/s12939-021-01464-9

**Published:** 2021-05-08

**Authors:** Haneen Shibli, Limor Aharonson-Daniel, Paula Feder-Bubis

**Affiliations:** 1 School of Public Health, Faculty of Health Sciences, Faculty of Health Sciences, PREPARED Center for Emergency Response Research, Ben-Gurion University of the Negev, Beer-Sheva, Israel; 2 Department of Health Systems Management, Faculty of Health Sciences & Guilford Glazer Faculty of Business and Management, Ben-Gurion University of the Negev, Beer-Sheva, Israel

**Keywords:** Healthcare accessibility, Arab, Bedouins, Women, Minority, Intersectionality, Cultural competency, Qualitative research

## Abstract

**Background:**

Access to healthcare services has major implications for vulnerable populations’ health. Socio-cultural and gender characteristics shape the utilization and access of healthcare services among ethnic minorities worldwide. One such vulnerable ethnic minority is the Arab Bedouin women in Israel. As women, they are marginalized in their community, where women do not have full equity and they experience multiple barriers to healthcare services The main objective of this study is to provide a nuanced, experiential, emic description of healthcare accessibility issues among Bedouin women in Israel. Identifying the barriers, they face in accessing healthcare may help healthcare policymakers make changes based on and tailored to Bedouin women’s needs.

**Methods:**

A qualitative study included in-depth semi-structured interviews with 21 Arab Bedouin village residents, consisting of 14 women and 7 men. Eligible participants were Arab Bedouins over 18 years of age and who used healthcare services at least once in the last 5 years. The interviews were carried out in Arabic-Bedouin dialect and included demographic questions, open-ended questions that asked about participants’ perceptions regarding their experiences with healthcare services, including the factors that helped and hindered them in accessing these services and questions regarding suggestions for improving the accessibility of healthcare services based on the identified needs. Data collected were analyzed using thematic analysis. Study trustworthiness was ensured using audit, reflexivity, and peer debriefing.

**Results:**

Arab Bedouin women experienced varied barriers while accessing healthcare services. This study uncovered how language, cultural and gender barriers intersect with other disadvantages ingrained in social norms, values and beliefs and affect the access of a minority women subgroup to healthcare services. The participants identified subgroups of Bedouin women (e.g. elderly Bedouin women) affected differently by these barriers.

**Conclusion:**

Taking into consideration the identified needs and the Arab Bedouin women’s unique characteristics, along with adopting the intersectional approach should help improve access to healthcare services among such a vulnerable subgroup and other subgroups within minorities worldwide.

## Introduction

Over the years, numerous variations persist in the definitions and conceptualizations of healthcare accessibility (HA). In the early 1990s, it was described as the level of adjustment between the characteristics of the healthcare resources and those of the population seeking and obtaining care [1]. This definition has been extended to include the actual use of services, and the reliance on and effectiveness of these services. Evaluating access now involves a clear emphasis on the characteristics of both the users and the services [2]. Although the definition of access to healthcare may vary, it generally includes the following dimensions: acceptability (i.e. beliefs about health, psychological, racial, cultural and ethnic factors determining the possibility that patients will accept services), affordability (i.e. socioeconomic status, monetary costs, ability to pay), availability, geographical accessibility and accommodation of services (i.e. appointment system, hours of operation, walk-in facilities) [3, 4].

Up to now, a number of studies have examined the barriers to healthcare services (HS) worldwide [5–10]. The major barriers to HS include financial barriers such as medications costs and insurance coverage [11], geographical barriers such as lack of public transportation and travel distance [12] and gender barriers. Access to HS was found to vary from one country to another due to the differences between health systems in those countries [5, 13]. Yet, studies have pointed out that the lack of HS leads to delay or forgoing needed healthcare and might negatively affect individuals’ health [11, 14]. Gaps in access to HS occur due to socioeconomic status, gender, ethnicity and more [15–17]. Vulnerable populations and minority groups are at higher risk to be affected by the lack of HA. More recent attention has focused on access to HS among disadvantaged populations such as elderly people [18–21], women [15, 22–24] and ethnic minority groups [25–28]. Access to HS becomes more complex when it comes to a population group that belongs to the three mentioned groups. The elderly Arab Bedouin women in Israel, a vulnerable population that is a minority within a minority, would be a good example to explain this complexity.

The Arab Bedouin women are part of an ethnic minority within the Arab Muslim minority population living in the Negev (Al-Naqab in Arabic), the desert located in southern Israel. According to the Israel Central Bureau of Statistics, approximately 268,867 Bedouin citizens live in the Negev, which comprises approximately 3% of the total population in Israel [29]. Compared to other minority groups in Israel, the Bedouin population is characterized by the lowest socioeconomic and educational levels and the highest levels of unemployment [30–32]. Almost 65% of the Bedouin population live in 11 recognized Bedouin townships and the rest of them live in unrecognized villages [33]. Sanitation in unrecognized villages is poor, without central waste or garbage disposal and lack of basic infrastructures such as water supply, electrical connections, public transportation, and access to HS. The social structure of the Bedouin community is traditional, tribal and patriarchal with men wielding most of the authority at home [34, 35]. An example of the marginalization of women as passive victims in the process of modernizing a traditional society is the dropout rate of Bedouin students from high schools [36], which reached a staggering 21.2% in 2016, significantly higher than the 7.3% among the general minority Arab group in Israel [37].

Bedouin women may be considered a doubly marginalized minority. As Bedouins, they are residents of the Israeli periphery and belong to a minority population; as women, they are marginalized in their community and are heavily affected by this marginalization [35]. Some Bedouin women marry at a young age in prearranged marriages within their clan. After marriage, some of them are subordinate to their husbands, doing the daily chores and bringing up their children. In some families, women are not allowed to leave their houses without a male chaperone. This social norm might interfere with the timely utilization of HS. Although illegal in Israel, it is estimated that 20% of Bedouin marriages are polygamous [34]. Indeed, several studies have shown that polygamous marriages have increased in the last 20–30 years [38, 39], specifically among young and well-educated men [34]. Previous studies have assessed the impact of polygamy on the physical and mental health, social and family functioning of women and children. Bedouin women living in polygamous marriages report higher rates of depression, anxiety, somatization, and less self-esteem and satisfaction with life compared to those in monogamous families [40–42].

The affordability of healthcare is seemingly irrelevant since Bedouin women are entitled to the basket of health services stipulated in the Israeli National Health Insurance Law [43]. However, the limited availability of medical services in the rural area of the Negev, and limitations of geographic mobility due to lack of transportation, and language and socio-cultural barriers prevent Bedouin women from obtaining appropriate health services [23, 44, 45]. A suitable solution to overcome the mentioned barriers is to establish community healthcare centres in the Bedouin villages and towns. Previous research has demonstrated the positive impact of establishing family health clinics in Bedouin villages on the accessibility of antenatal care. The establishment of these centres improved the ability of these women to access HS independently and reduced additional barriers to HS. However, the limited hours of operation and lack of adequate staff interfered with the proper functioning of these centers [46].

The mentioned Individual characteristics along with the social and environmental characteristics are part of the factors that shape the access to HS and health of Bedouin women, which are known as social determinants of health [47]. Understanding the effect of social determinants of health on minority groups, regarding access to HS, requires an in-depth look at how these factors combine and influence, not by a single axis but by many axes and shape the access to HS. A suggested way of understanding this complexity is using the framework of intersectionality. Thus far, the emerging intersectionality approach has evolved as a feminist approach [48] and has been gradually used in several fields. In the health field, López et al. [49], studied the social determinants of health in the lens of intersectionality and used it to reduce health disparities, and promote health equity and social justice. A qualitative study conducted among vulnerable individuals in Kenya, applied the intersectionality lens to examine how power relations intersect to produce vulnerabilities for specific groups. The researchers identified social dimensions such as gender, age, poverty and geographic location that intersect, contribute to health vulnerability and shape the health utilization of individuals [50]. Studies of women’s health also adapted the intersectionality as a framework to address social inequality, health disparities and influence on physical and mental health among certain groups of women [51–53]. In view of the intersection of living conditions, social and cultural norms, religious values, social networks and interpersonal relationships, health services and gender – i.e., the various layers of social determinants of health, the goal of this study was to provide a nuanced, experiential, emic description of HA issues among Bedouin women. Identifying the barriers, they face in accessing healthcare may help healthcare policymakers make changes based on and tailored to Bedouin women’s needs.

## Methods

### Study’s design and population

A qualitative approach was used to obtain an understanding of Bedouin women’s perceptions regarding their access to HS. This approach seeks to study the experiences of patients and help to identify potentially modifiable factors for improving health care [54]. Our study included face-to-face, in-depth, semi-structured interviews with 21 Bedouin village residents. To ensure maximum variation sampling, we recruited Bedouin residents with varied demographic characteristics. Eligible participants were Bedouins over 18 years of age, who lived in one of the Bedouin villages in the northern Negev region and had used one of the health services available in this region at least once in the last 5 years. Fourteen women and seven men were recruited with the help of students, educators and nurses from the Bedouin population. None of the participants had any prior familiarity with the interviewer before they participated in the study, and they signed an informed consent form. Participation was voluntary, and participants could withdraw from the interview at any time if they chose to do so.

### Data collection

Data were collected between April and August 2016. Interviews took place at locations convenient for each participant, mostly in the women’s homes and the men’s workplaces, based on the preference of the participants themselves. The interviews lasted 45–60 min and were conducted in an Arabic-Bedouin dialect by the first author. An interview guide to identifying barriers to and facilitators of access to healthcare and to map the perceptions of Bedouins about their healthcare experiences was developed. The interview guide allowed flexibility regarding the order in which the questions were asked, and which questions and clarification questions were asked when needed, providing a rich understanding of the participants’ thoughts and opinions [55, 56].

Each interview began with general demographic questions (i.e., gender, age, marital status, number of children, education level, the form of settlement, socioeconomic status, and Hebrew proficiency) to become familiar with the participants and to build rapport with them. We then asked open-ended questions on topics related to the participants’ perceptions about and experiences with HS, including the factors that helped and hindered them in accessing these services. The last part of the interview asked the interviewees for their suggestions regarding improving the supply and accessibility of HS based on their own identified needs. We took notes during the interviews. The interviews were audiotaped, and subsequently, translated and transcribed verbatim. Data collection continued until thematic saturation was achieved.

### Data analysis

Data coding and analysis occurred simultaneously with data collection. We read and reread the interviews repeatedly to gain familiarity with the data. Recurrent ideas were identified in this initial stage. Peer debriefing sessions were held after each interview by the research team to analyze the data, following thematic analysis principles [57]. Initial codes were generated and a thematic map including the codes and themes was developed. We then reviewed the themes and named them. Additionally, we met to discuss and review the findings on an ongoing basis, and to engage in reflexive analysis. To ensure the participants’ anonymity, we replaced their names with numbers and omitted any identifying details.

## Results

### Participants’ characteristics

Table 1 lists the participants’ socio-demographic characteristics. The participants’ age ranged from 19 to 70 years and the mean age was 38.2 ± 14.1 years old. Most of the participants lived in recognized villages and reported income typical of low socioeconomic status in the country.

**Table 1:** Sociodemographic characteristics of the study’s population

Participant #	Gender	Age	Marital status	Form of settlement	Education level	Socioeconomic status	Hebrew proficiency^a^
1	Male	55	Married	Recognized village	Academic degree	Above average	Very good
2	Female	20	Single	Recognized village	High school	Low	Good
3	Female	19	Single	Unrecognized village	High school	Low	Poor
4	Female	23	Single	Recognized village	Academic degree	Low	Very good
5	Female	44	Married	Recognized village	High school	Low	Very good
6	Female	40	Married	Recognized village	Partial high school	Low	Poor
7	Female	70	Widowed	Recognized village	No formal education	Low	None
8	Female	21	Single	Recognized village	High school	Low	Very good
9	Female	44	Married	Recognized village	Academic degree	Average	Very good
10	Female	56	Married	Recognized village	No formal education	Low	None
11	Female	49	Married	Recognized village	Partial high school	Low	Poor
12	Male	30	Married	Recognized village	No formal education	Low	Good
13	Male	31	Married	Recognized village	Academic degree	Low	Very good
14	Male	25	Single	Recognized village	Academic degree	Average	Very good
15	Female	25	Married	Recognized village	Academic degree	Average	Very good
16	Male	48	Married	Unrecognized village	Academic degree	Average	Very good
17	Male	36	Married	Recognized village	Academic degree	Average	Very good
18	Male	45	Married	Recognized village	Academic degree	Average	Very good
19	Female	37	Married	Recognized village	Academic degree	Average	Very good
20	Female	55	Married	Recognized village	No formal education	Low	None
21	Female	30	Single	Unrecognized village	Partial high school	Low	Poor

^a^According to the participant’s self-report

### Accessibility barriers among Bedouins in the northern Negev region

Three themes emerged from the analysis of the perceptions of the Bedouin population about the accessibility of HS: the language barrier, cultural competency, and gender-based barriers. Surprisingly, no differences were found regarding perceptions on healthcare access barriers among both genders and participants from recognized and unrecognized villages. All the participants echoed what they saw or experienced regarding access barriers that women and residents of unrecognized villages face.

#### The language barrier

The language barrier was described as miscommunication between healthcare providers and patients as a result of speaking different languages and dialects. Many of the participants stated that the language barrier affects access to HS, particularly among elderly Bedouin women. A young participant explained:

Together with the difficulties in understanding the (Hebrew) language, there was a complaint about elderly women coming to the primary healthcare clinics and waiting for the doctors for many hours because they couldn’t understand that they were called via an automatic healthcare queue management system in the waiting rooms. (Participant 4, Female)

Some Bedouin women who visited primary healthcare clinics did not understand Hebrew, which is the language used by the majority of the Jewish population, including administrative personnel and physicians who worked in these clinics. Moreover, one participant stated with anger and frustration that even if non-Arab physicians wanted to communicate better with Bedouin patients, they would still face a language barrier because the Arabic-Bedouin dialect differs from the Arabic commonly spoken in the country: “Many physicians from different backgrounds are here (in the clinics), Jewish physicians, Russians as well. Let’s say if the physicians want to speak in Arabic (with patients), they can’t. No way!” (Participant 17, Male).

Miscommunication often forced the Bedouin patients to ask for help from someone who spoke both languages: “Unfortunately, no one works in the clinic and whose job is to help; you should always ask for help.” (Participant 2, Female). Many might regard asking for help in such situations as natural. However, some Bedouin women would be embarrassed to actively ask for assistance because doing so is regarded as culturally inappropriate and unacceptable.

In certain cases, women’s privacy was compromised because they had to communicate through someone else when speaking with their physician. Moreover, those providing the services had to rely on the interpretations of non-professionals because the Bedouin women could not ask for appropriate treatment themselves. This situation could lead to the skewing of the doctor’s counsel because the frankness of the patients’ report may have been compromised by the need to use a mediator and the fact that these translators were less knowledgeable about medical terminology.

Each patient has (the right to) privacy, especially among our Bedouin community. For example, a 17-year-old female patient who wants to talk about her personal health issue to a male doctor, and there’s also a translator in the room... It’s a problem. She can hardly talk to the doctor. So, imagine how she feels when there’s also a (male) translator in the room. (Participant.18, Male)

However, the majority of participants unequivocally pointed to the need for translation and interpreter services for Bedouin patients in the healthcare system: “The best solution, in my opinion, is to have (in these clinics) physicians from the Bedouin population who speak the language or a translator or a Bedouin woman in the (clinic’s) office so that she can translate in urgent cases.” (Participant 17, Male). While the majority of participants agreed with the translation services as a solution for overcoming the language barrier, a few other interviewees considered it an unsatisfactory solution. Instead, they argued for a permanent, structural solution, one that accommodates social and gender norms.

One participant suggested a creative solution for analphabetic Bedouin women and elderly people in order to overcome the language barrier:

The solution for this (the language barrier) is a screen that displays the numbers in a very large size and then women who could not read and write … and elderly people... will get the number and look at the screen, if the number on the screen is the same as the number they got on this ticket, it means that now it is their turn to enter the room and see the doctor … (Participant 4, Female)

It is important to note that queue management systems were available in both languages, Hebrew, and Arabic, in some of the HS and were used by HMOs in Israel to help patients in those clinics.

#### Cultural competency

The interviews reflect a lack of cultural competency in HS and among healthcare providers, even Arab ones, vis-a-vis the Bedouin population. For example, an elderly woman, who spoke enthusiastically about an Arab nutritionist from the Northern region of the country who was unfamiliar with the traditional lifestyle and eating habits of elderly Bedouin women, reported that “She talked to us and explained about herbs and the damage they cause … You have to use fruits, like bananas, apples, cherries, and put them in a blender.” (Participant 7, Female). The Arab nutritionist addressed the Bedouin women in their language, and it was highly appreciated by the Bedouin women. Yet the content was completely irrelevant, and the advice provided discredited the widely accepted use of herbs and ignored the fact that there is no water and electricity in some of the Bedouin villages in the northern Negev region. Furthermore, there is no tropical fruit in the middle of the desert, making her recommendations impractical.

The lack of cultural competence was also evident in the format in which HS were provided. A recurrent topic was that the community clinics’ visiting hours were unsuitable for Bedouin women’s lifestyle and cultural norms. A female Bedouin participant explained that “There are official visiting hours in the evening. They (Bedouin women) can’t come late in the evening. It’s hard for them to come at these hours.” (Participant 6, Female). A Bedouin woman is usually unable to leave her home during evening hours because she is expected to do household chores and attend to her husband and children.

In several cases, Bedouin patients reported seeking private healthcare in the Palestinian Authority as a way of overcoming the language and cultural barriers in the Israeli system “I went to see a doctor in Dhahiriya (in the Palestinian Authority)... He treated me and made me feel better... I did the same medical tests and the treatment in Dhahiriya is the same as the one in Israel” (Participant 6, Female). These physicians are well known and familiar with the Bedouin community, and their personal and professional treatment is viewed as satisfactory. The description that their treatment is “the same as the one in Israel” is a justification, since these clinicians are unregistered and unrecognized by the Israeli healthcare system. Thus, the continuity of care of Bedouin patients is compromised in both systems.

Some participants mentioned that in order to overcome the cultural competency barrier, they turned to traditional practices such as asking for help from their local community leaders and heads of tribes: “Women in my tribe turn to the Sheikh or their neighbor or someone from the tribe and the problems are solved. This way we help the system as we solve our problems ourselves.” (Participant 16, Male). The social structure and tribal cohesion in the Bedouin community, together with familiarity and trusted relationships with the community leaders, encourage women to ask for their help. Here again, this practice also affects the continuity of their care in the health system. The gender-based barrier.

One of the major challenges for Bedouin women was the insensitivity of HS providers to Bedouin-Muslim gender norms. Many young women reported they faced situations similar to the following account: “In certain situations, I am embarrassed to go see a male doctor and tell him what I have because he isn’t a woman. It prevents me from making a doctor’s appointment and feeling comfortable discussing my health problems.” (Participant 2, Female). In this patriarchal society, gender concordance between the healthcare provider and the patient is critical because of socio-cultural and religious practices. The embarrassment prevents Bedouin women from speaking to their doctors or exposing their bodies to male doctors. Indeed, this issue was reported to be one of the most common reasons why Bedouin women delay or forgo seeking treatment.

Gender norms among the Bedouin community also shape the perceptions of the treatment given to patients. The seemingly trivial prescription of eyeglasses has particular social connotations in the Bedouin community. A young woman explained her reasons for her resentment about being prescribed glasses:

People don’t use healthcare services for different reasons, if someone needs glasses, for example, the society around me will start mocking me, saying ‘You wear glasses because you are blind.’ (So) I wear glasses depending on where I am; I don’t wear them all the time. In our case, as a young Bedouin woman, if I wear glasses, they will say I am blind. So, who would propose to me? (Participant 4, Female)

Marriage is a central value for Bedouin women, giving them a high social status in the community. Wearing eyeglasses carries a stigma in Bedouin society, reducing a woman’s chances of getting married.

An interviewee from one of the unrecognized Bedouin villages stated that the lack of physical accessibility and gender-based barriers exacerbated the difficulties and widened the gaps between Bedouin men and women in the utilization of HS:

Not everyone owns a car at home... To get from the unrecognized village to the nearest healthcare clinic outside the village, he has to catch a ride with other men from the community that are on the way to their workplace... Only Bedouin men can do this. Women can’t. (Participant 3, Female)

For Bedouin women, social and gender-based norms prevent catching a ride with a male stranger. The difficulty or even impossibility of getting from an unrecognized Bedouin village to the nearest clinic without a private car or public transportation (unavailable in unrecognized villages) takes a heavy toll, especially on women. Getting to a clinic could mean a full day endeavor for Bedouin women.

One young Bedouin woman highlighted other issues such as customs and religious practices, including wearing a hijab, while receiving treatment:

Of course, I’m tired and I don’t wear my hijab during hospitalization. Men go in and out, and sometimes people accidentally open the hospital curtain. With my sickness and weakness, I couldn’t close the curtain because I’m tired... I had to deal with many other things beyond my health problem. (Participant 8, Female)

The participants, in general, agreed that the combination of these obstacles had the strongest impact on young Bedouin women of childbearing age and elderly Bedouin women, who were perceived as especially vulnerable groups in the Bedouin community.

## Discussion

To our knowledge, this is one of a few qualitative studies regarding healthcare accessibility among the Bedouin population that includes both Bedouin women and men participants. We included both genders to obtain a comprehensive view of healthcare barriers from both perspectives.

The language barrier we identified accords with the findings of earlier studies among different minorities around the world [27, 58–60] and other local studies among Bedouin women in Israel [44, 45]. Communication is a basic requirement for the implementation of patient-centered care and plays an important role in the use of HS. However, non-Arab as well as Arab healthcare providers do not speak the Arab-Bedouin dialect, and elderly Bedouin, in particular, have little Hebrew proficiency. Despite the existence of a national health insurance law that provides HS to all, the miscommunication between the non-Bedouin clinical staff and Bedouin patients reduces the uptake of the content of HS and the ability to communicate during treatment.

Lack of cultural competency is also a considerable barrier when accessing HS. Numerous studies have demonstrated the importance of cultural understanding and competency by healthcare providers for the health outcomes of minority populations [61, 62]. The movement toward cultural competence in healthcare has gained national attention and is now recognized by healthcare policymakers [63]. However, providing cultural competence programs in healthcare is insufficient. Cultural competence must occur at all levels, within layers and subgroups of the community, as defined by the NIH [64]. The quote of the nutritionist who provided counselling to an elderly Bedouin woman is a clear example of how sharing the common native Arabic language does not necessarily imply cultural competence.

Another unanticipated finding is the differentiation between sub-groups of Bedouin women when trying to access HS. Our results are consistent with Queder’s (2007) findings that showed differences and diversity among subgroups within Bedouin women. However, we documented that the barriers we identified affected mainly Bedouin women from unrecognized villages, elderly women and young Bedouin women of childbearing age. Our findings support previous research [65] demonstrating that lack of gender concordance between the healthcare provider and the patients leads Bedouin women of childbearing age to delay seeking healthcare. Moreover, Bedouin women from unrecognized villages face gender-based barriers and a lack of physical accessibility due to the lack of basic infrastructures in their villages. Elderly women face all of these issues, compounded by their low level of literacy about healthcare and the difficulty they have navigating the healthcare system.

The northern Negev population that seeks treatment and utilizes HS outside the Israeli healthcare system is an overlooked, yet important phenomenon. Despite their low socioeconomic status, Bedouin women tend to use private HS in the Palestinian Authority where they find healthcare workers who understand their culture and lifestyle. In some cases, there is an intersection between culture and gender-based barriers. For example, most Bedouin husbands require that they accompany women when they go to the clinic, so they can be informed about and in control of the health status of their wives. However, the Israeli Patient’s Rights Bill [43] makes this request unacceptable for Israeli healthcare providers. Thus, seeking care in the Palestinian Authority seems to be a mechanism that meets women’s health needs while maintaining the husbands’ primacy over their spouses’ health status.

Our results can be understood in light of intersectionality theories [48, 66, 67]. The respondents’ answers emphasize that access to HS is not shaped by only one factor. In line with Collins (2000) [66], it is possible to interpret the results as an expression of how gender, socio-cultural hierarchies, and power interact and affect Bedouin women’s access to HS. A multi-level intersectional approach [48] may be instrumental in finding culturally sound ways to resolve these issues.

Gender and cultural awareness of all sub-groups would help ensure that HS are accessible and offer universal coverage. Healthcare policymakers need to work with the community, consider the characteristics of both the users, providers and services, and adopt practical measures that accord with gender and cultural norms, social expectations and the users’ identified needs to improve the acceptability, affordability, availability and accommodation of HS among Bedouin women and vulnerable subgroups within minorities. Indeed, such an approach is important for all government and civilian bodies when dealing with minorities.

Despite its contributions, the study has some limitations. The interviews were conducted in the Arabic-Bedouin dialect and then translated into English. Such a process might result in the loss of cultural context and nuances that the translators and analysts missed [68, 69]. To cope with this issue, the first author, who also conducted the interviews, translated the texts to ensure retaining the participants’ intended meaning. Moreover, the sample might not represent all sectors of the Bedouin society since most of the participants were relatively highly-educated and from recognized villages.

### Conclusion and implications for practice and/or policy

Looking through an intersectional lens allows us to highlight the invisible and characterize the differently affected subgroups among a vulnerable population. Therefore, our findings based on the experiences of women from the same minority group emphasize that “one size does not fit all” which means that targeting a vulnerable population is not sufficient [70]. To reduce the gaps and improve access, healthcare services should be culturally sensitive and tailored to the specific needs of groups among minorities such the Bedouin women. Additionally, all subgroups among Bedouin must be considered and served based on their specific needs. Additional investment could help in developing, evaluating, and supporting effective healthcare delivery models designed to meet the specific needs of vulnerable populations within the Bedouin women. More broadly, a combination of such strategies is likely necessary for healthcare provision for minority women and vulnerable populations worldwide.

## Data Availability

The datasets used and/or analyzed during the current study are available from the corresponding author.

## References

[1] Frenk J, White KL. The concept and measurement of accessibility. PAHO Sci Publ. 1992;27:842–55.

[2] Levesque J-F, Harris MF, Russell G (2013). Patient-centred access to health care: conceptualising access at the interface of health systems and populations. Int J Equity Health.

[3] Gulliford M, Figueroa-Munoz J, Morgan M, Hughes D, Gibson B, Beech R (2002). What does’ access to health care’mean?. J Health Serv Res Policy.

[4] Penchansky R, Thomas JW (1981). The concept of access: definition and relationship to consumer satisfaction. Med Care JSTOR.

[5] Garcia-Subirats I, Vargas I, Mogollón-Pérez AS, De Paepe P, da Silva MRF, Unger JP (2014). Barriers in access to healthcare in countries with different health systems. A cross-sectional study in municipalities of Central Colombia and North-Eastern Brazil. Soc Sci Med.

[6] Corscadden L, Levesque JF, Lewis V, Strumpf E, Breton M, Russell G (2018). Factors associated with multiple barriers to access to primary care: an international analysis. Int J Equity Health.

[7] Douthit N, Kiv S, Dwolatzky T, Biswas S (2015). Exposing some important barriers to health care access in the rural USA. Public Health.

[8] Jacobs B, Ir P, Bigdeli M, Annear PL, Van Damme W (2012). Addressing access barriers to health services: an analytical framework for selecting appropriate interventions in low-income Asian countries. Health Policy Plan.

[9] Peters DH, Garg A, Bloom G, Walker DG, Brieger WR, Hafizur RM (2008). Poverty and access to health care in developing countries. Ann N Y Acad Sci.

[10] Harris B, Goudge J, Ataguba JE, McIntyre D, Nxumalo N, Jikwana S, Chersich M (2011). Inequities in access to health care in South Africa. J Public Health Policy.

[11] Wang T-F, Shi L, Nie X, Zhu J (2013). Race/ethnicity, insurance, income and access to care: the influence of health status. Int J Equity Health.

[12] Syed ST, Gerber BS, Sharp LK (2013). Traveling towards disease: transportation barriers to health care access. J Community Health.

[13] Macinko J, Cristina Drumond Andrade F, Bof de Andrade F, Lima-Costa MF (2020). Universal health coverage: are older adults being left behind? Evidence from aging cohorts in twenty-three countries: study examines access to care, use, catastrophic expenditures, and other factors among several aging cohorts in twenty-three countries. Health Aff.

[14] Alzubaidi H, McNamara K, Browning C, Marriott J (2015). Barriers and enablers to healthcare access and use among Arabic-speaking and Caucasian English-speaking patients with type 2 diabetes mellitus: A qualitative comparative study. BMJ Open.

[15] Floyd A, Sakellariou D (2017). Healthcare access for refugee women with limited literacy: layers of disadvantage. Int J Equity Health.

[16] Canedo JR, Miller ST, Schlundt D, Fadden MK, Sanderson M (2018). Racial/ethnic disparities in diabetes quality of care: the role of healthcare access and socioeconomic status. J Racial Ethn Heal disparities.

[17] Filc D, Davidovich N, Novack L, Balicer RD (2014). Is socioeconomic status associated with utilization of health care services in a single-payer universal health care system?. Int J Equity Health.

[18] Suurmond J, Rosenmöller DL, el Mesbahi H, Lamkaddem M, Essink-Bot ML (2016). Barriers in access to home care services among ethnic minority and Dutch elderly - a qualitative study. Int J Nurs Stud.

[19] Thorpe JM, Thorpe CT, Kennelty KA, Pandhi N (2011). Patterns of perceived barriers to medical care in older adults: a latent class analysis. BMC Health Serv Res.

[20] Goins RT, Williams KA, Carter MW, Spencer SM, Solovieva T (2005). Perceived barriers to health care access among rural older adults: a qualitative study. J Rural Heal.

[21] Wandera SO, Kwagala B, Ntozi J (2015). Determinants of access to healthcare by older persons in Uganda: a cross-sectional study. Int J Equity Health.

[22] Tackett S, Young JH, Putman S, Wiener C, Deruggiero K, Bayram JD. Barriers to healthcare among Muslim women: a narrative review of the literature. Womens Stud Int Forum. 2018;69:190–4.

[23] Alfayumi-Zeadna S, Froimovici M, Azbarga Z, Grotto I, Daoud N (2019). Barriers to postpartum depression treatment among indigenous Bedouin women in Israel: a focus group study. Health Soc Care Community.

[24] Janevic T, Sripad P, Bradley E, Dimitrievska V (2011). “There’s no kind of respect here” A qualitative study of racism and access to maternal health care among Romani women in the Balkans. Int J Equity Health.

[25] Tang K, Zhao Y, Li B, Zhang S, Lee SH. Health inequity on access to services in the ethnic minority regions of Northeastern Myanmar: a cross-sectional study. BMJ Open 2017;7(12):1–8. 10.1136/bmjopen-2017-017770 PMC5735411 29247090

[26] Colombini M, Rechel B, Mayhew SH (2012). Access of Roma to sexual and reproductive health services: qualitative findings from Albania, Bulgaria and Macedonia. Glob Public Health.

[27] Memon A, Taylor K, Mohebati LM, Sundin J, Cooper M, Scanlon T, et al. Perceived barriers to accessing mental health services among black and minority ethnic (BME) communities: a qualitative study in Southeast England. BMJ Open. 2016;6:1–9. 10.1136/bmjopen-2016-012337 PMC5128839 27852712

[28] Chen J, Vargas-Bustamante A, Mortensen K, Ortega AN (2016). Racial and ethnic disparities in health care access and utilization under the Affordable Care Act. Med Care.

[29] Israel Central Bureau of Statistics (2019). Population by district, sub-district and religion.

[30] Abu-Rabia A (2000). Employment and unemployment among the Negev Bedouin. Nomad People JSTOR.

[31] Israel Central Bureau of Statistic (2019). Localities within regional councils by name of regional of the socio-economic index.

[32] Israel Central Bureau of Statistic (2018). Arabs aged 15 and over, by labour force characteristics, type of locality of residence, district and sub-district of residence, religion and sex.

[33] The Knesset Research and Information Center (2013). Arrangement of Bedouin Settlement in the Negev.

[34] Daoud N, Shoham-Vardi I, Urquia ML, O’Campo P (2014). Polygamy and poor mental health among Arab Bedouin women: do socioeconomic position and social support matter?. Ethn Health.

[35] Queder SA-R (2007). Permission to rebel: Arab Bedouin women’s changing negotiation of social roles. Fem Stud JSTOR.

[36] Abu-Rabia-Queder S (2006). Between tradition and modernization: understanding the problem of female Bedouin dropouts. Br J Social Educ.

[37] Eyal Y, King J, Frankel M & TO. The program to promote economic growth and development for the Bedouin population in the Negev, Government Resolution 3708. 2018;RR–772–18.

[38] Abu-Rabia A, Elbedour S, Scham S (2008). Polygyny and post-nomadism among the Bedouin in Israel. Anthropol Middle East. Berghahn J.

[39] Dinero SC (2012). Neo-polygamous activity among the Bedouin of the Negev, Israel: dysfunction, adaptation—or both?. J Comp Fam Stud.

[40] Al-Krenawi A (1999). Women of polygamous marriages in primary health care centers. Contemp Fam Ther.

[41] Al-Krenawi A, Graham JR (2006). A comparison of family functioning, life and marital satisfaction, and mental health of women in polygamous and monogamous marriages. Int J Soc Psychiatry.

[42] Al-Krenawi A, Slonim-Nevo V (2008). Psychosocial and familial functioning of children from polygynous and monogamous families. J Soc Psychol.

[43] The Knesset (1994). The National Health Insurance law.

[44] Daoud N, O’Campo P, Anderson K, Agbaria AK, Shoham-Vardi I (2012). The social ecology of maternal infant care in socially and economically marginalized community in southern Israel. Health Educ Res.

[45] Daoud N, Shoham-Vardi I (2015). Maternal perceptions of social context and adherence to maternal and child health (MCH) clinic recommendations among marginalized Bedouin mothers. Matern Child Health J.

[46] Gottlieb N, Belmaker I, Bilenko N, Davidovitch N (2011). Bedouin-Arab women’s access to antenatal care at the interface of physical and structural barriers: a pilot study. Glob Public Health.

[47] World Health Organization. Social determinants of health. Regional Office for South-East Asia; 2008.

[48] Crenshaw K. Demarginalizing the intersection of race and sex: A black feminist critique of antidiscrimination doctrine, feminist theory and antiracist politics. u Chi Leg f. HeinOnline; 1989;139.

[49] López N, Gadsden VL (2016). Health inequities, social determinants, and intersectionality.

[50] McCollum R, Taegtmeyer M, Otiso L, Tolhurst R, Mireku M, Martineau T (2019). Applying an intersectionality lens to examine health for vulnerable individuals following devolution in Kenya. Int J Equity Health.

[51] Hankivsky O, Reid C, Cormier R, Varcoe C, Clark N, Benoit C (2010). Exploring the promises of intersectionality for advancing women’s health research. Int J Equity Health.

[52] Lewis JA, Williams MG, Peppers EJ, Gadson CA (2017). Applying intersectionality to explore the relations between gendered racism and health among Black women. J Couns Psychol.

[53] Daoud N, Ali Saleh-Darawshy N, Gao M, Sergienko R, Sestito SR, Geraisy N (2019). Multiple forms of discrimination and postpartum depression among indigenous Palestinian-Arab, Jewish immigrants and non-immigrant Jewish mothers. BMC Public Health.

[54] Wright EB, Holcombe C, Salmon P (2004). Doctors’ communication of trust, care, and respect in breast cancer: qualitative study. Br Med J.

[55] Rubin HJ, Rubin IS. Qualitative interviewing: the art of hearing data. SAGE Publications; 2011.

[56] Berg BL, Lune H, Lune H (2004). Qualitative research methods for the social sciences.

[57] Braun V, Clarke V (2006). Using thematic analysis in psychology. Qual Res Psychol.

[58] De Moissac D, Bowen S (2019). Impact of language barriers on quality of care and patient safety for official language minority Francophones in Canada. J Patient Exp.

[59] Hannan J (2015). Minority mothers’ healthcare beliefs, commonly used alternative healthcare practices, and potential complications for infants and children. J Am Assoc Nurse Pract.

[60] Ngwakongnwi E, Hemmelgarn BR, Musto R, Quan H, King-Shier KM (2012). Experiences of French speaking immigrants and non-immigrants accessing health care services in a large Canadian city. Int J Environ Res Public Health.

[61] Belintxon M, de Dicastillo López O. The challenges of health promotion in a multicultural society: a narrative review. An Sist Sanit Navar. 2014;37(3):401–9. 10.4321/s1137-66272014000300009 25567393

[62] Govere L, Govere EM (2016). How effective is cultural competence training of healthcare providers on improving patient satisfaction of minority groups? A systematic review of literature. Worldviews Evid -Based Nurs.

[63] Napier D, Depledge MH, Knipper M, Lovell R, Ponarin E, Sanabria E, et al. Culture matters: using a cultural contexts of health approach to enhance policy-making. World Health Organization Regional Office for Europe; 2017.

[64] National Institutes of Health (2017). Cultural respect.

[65] Padela AI, Gunter K, Killawi A, Heisler M (2012). Religious values and healthcare accommodations: voices from the American Muslim community. J Gen Intern Med.

[66] Collins PH (2000). Gender, black feminism, and black political economy. Ann Am Acad Pol Soc Sci.

[67] Cho S, Crenshaw KW, McCall L (2013). Toward a field of intersectionality studies: Theory, applications, and praxis. Signs J Women Cult Soc.

[68] Squires A (2009). Methodological challenges in cross-language qualitative research: a research review. Int J Nurs Stud.

[69] Venuti L (2009). Translation, intertextuality, interpretation. Roman Stud.

[70] Kreuter MW, Lukwago SN, Bucholtz DC, Clark EM, Sanders-Thompson V (2003). Achieving cultural appropriateness in health promotion programs: targeted and tailored approaches. Heal Educ Behav.

